# Accuracy and cut-off values of different acoustic measures in the sustained vowel task of Brazilian Portuguese speakers

**DOI:** 10.1590/2317-1782/e20250288en

**Published:** 2026-07-03

**Authors:** Amanda Feliciano, Mara Behlau, Jordana Balbinot, Samuel Ribeiro de Abreu, Vanessa Veis Ribeiro, Leonardo Wanderley Lopes

**Affiliations:** 1 Centro de Estudos da Voz – CEV - São Paulo (SP), Brasil.; 2 Universidade Federal de Rondonópolis – UFR - Rondonópolis (MS), Brasil.; 3 Departamento de Fonoaudiologia, Faculdade de Ciências e Tecnologias em Saúde, Universidade de Brasília – UnB - Brasília (DF), Brasil.; 4 Departamento de Fonoaudiologia, Centro de Ciências da Saúde, Universidade Federal da Paraíba – UFPB - João Pessoa (PB), Brasil.

**Keywords:** Voice, Acoustic, Voice Disorders, Dysphonia, Diagnostic

## Abstract

**Purpose:**

To define cut-off values for acoustic measures extracted from the sustained vowel [a] task to discriminate between the presence and absence of vocal disorders in Brazilian Portuguese speakers.

**Methods:**

The sample consisted of 376 participants, 288 female and 88 male, with a mean age of 41.20 ± 14.04 years. All individuals underwent a laryngological evaluation and a voice recording session, during which a sustained vowel [a] sample was collected. Subsequently both auditory-perceptual and acoustic analyses of the voice were performed. Among the participants 277 were classified as having a vocal disorder and 99 as having no vocal disorder. A total of 44 acoustic measures were extracted and analyzed using ROC curves to determine the cut-off value for each measure, along with their performance metrics (sensitivity, specificity and AUC).

**Results:**

Cut-off values were established for 23 of the 44 extracted acoustic measures. Six measures showed robust and clinically relevant performance in discriminating between voices with and without vocal disorders in Brazilian Portuguese speakers. The measures with AUC values greater than 0.70 and balanced sensitivity and specificity above 70% were: Amplitude Variability Index (AVI) (≤0,91), Glottal-to-noise excitation (GNE)2000Hz (>0,92), Spectral Noise Level (SNL)100-2600Hz (≤7,71), SNL100-3000Hz (≤3,38), SNL100-8000Hz (≤10,95) and SNL5100-8000Hz (≤–1,84).

**Conclusion:**

Six acoustic measures demonstrated satisfactory performance in identifying the presence of vocal disorders in Brazilian Portuguese speakers. Ten measures showed good performance for voice screening purposes and seven for diagnostic confirmation of vocal disorders.

## INTRODUCTION

The multidimensional voice assessment procedures include acoustic analysis, auditory-perceptual judgment (APJ), aerodynamic analysis, structural and vibratory examination of the larynx, and vocal self-assessment^(1)^. Acoustic analysis provides measures to quantify the characteristics of the vocal signal and perform a descriptive evaluation of the visual patterns of these signals^(2)^. This contributes to screening, diagnostic confirmation, and monitoring the effectiveness of therapeutic interventions in individuals with voice disorders^(3,4)^.

There are several acoustic measures for assessing the vocal signal. Among the conventional and most widely used measures are fundamental frequency (F0), jitter, shimmer, and harmonic-to-noise ratio (HNR)^(1)^. Conventional measurements are particularly effective in mild vocal deviations. Spectral and cepstral measurements, more widely used and studied in recent years, result from the double transformation of the acoustic voice signal, revealing the degree of harmonic organization in the spectrum. They are effective for analyzing deviated voices, as they are strong predictors of vocal deviation^(5)^.

The clinical applicability of acoustic measurements depends on the definition of cutoff values, with consistent diagnostic performance appropriate to the target population. Few acoustic measures have defined cutoff values in Brazilian Portuguese (BP), which limits intersubject comparison and comparison of the patient's voice with reference values^(6)^, in dissonance with well-founded and conscious clinical practice.

Recently, cutoff values ​​were established for two multiparametric indices, Acoustic Voice Quality Index (AVQI) and Acoustic Breathiness Index (ABI), to help identify vocal disorders in BP speakers^(7)^. With excellent diagnostic accuracy, the AVQI achieved a cutoff point of 1.33, sensitivity of 78.8%, and specificity of 90.6%. In turn, the ABI showed good diagnostic accuracy, with a cutoff point of 2.94, sensitivity of 75.3%, and specificity of 93.4%^(7)^. This highlights the strength of AVQI and ABI as objective tools for assessing and monitoring vocal disorders^(7,8)^.

Two other measures have stood out as objective parameters in vocal acoustic analysis, the *Cepstral Peak Prominence* (CPP) and the *Smoothed Cepstral Peak Prominence* (CPPS). They have a good correlation with APJ and high sensitivity in detecting vocal deviations, without depending on the extraction of the fundamental frequency. There are cutoff values ​​to discriminate between voices with and without vocal disorders in BP^(6)^. The study identified that values ​​below 28.15 decibels (dB) (vowel [ɛ]), 28.77 dB (vowel [a]) and 28.58 dB (Consensus Auditory-Perceptual Evaluation of Voice - CAPE-V sentence), and that for the CPPS, values ​​below 16.42 dB (vowel [ɛ]), 17.02 dB (vowel [a]), and 11.30 dB (all CAPE-V sentences) are indicative of a vocal disorder^(6)^.

The cutoff point is derived from the best relationship between sensitivity and specificity. It is essential to identify measures with balanced performance that simultaneously present satisfactory values ​​of sensitivity and specificity. These measures have the ability to correctly identify both individuals with vocal disorders (true positives) and individuals without the condition (true negatives), reducing the occurrence of false diagnoses. This balance is generally represented by area under the curve (AUC) values ​​greater than 0.70 in ROC analyses, indicating good discriminative performance^(1)^.

The classification regarding the presence or absence of specific health conditions depends on the accuracy of the measurement or testing procedure. This accuracy reflects the agreement between the findings of a new test, called an index test, and those obtained through the most reliable existing method for detecting the health outcome in question, the reference standard^(9)^. Therefore, selecting an appropriate reference standard is imperative to validate the accuracy metrics of an emerging diagnostic test. When choosing a reference test, clinicians face challenges in establishing criteria for vocal normality due to its inherently multidimensional nature. Currently, there is no single reference method capable of accurately differentiating vocally healthy individuals from those with a vocal disorder^(10)^. Given the lack of robust scientific evidence and expert consensus on the detection of certain health conditions, alternative reference standards must be developed^(9)^.

Although APJ and self-assessment are traditionally used as preliminary methods to identify voice disorders^(11)^, visual inspection of the larynx (including laryngoscopy and stroboscopy) is considered the gold standard for confirming the diagnostic categorization of voice disorders^(10)^. Synthesizing information from different procedures can construct an integrative reference criterion, derived from the correlation between the results of a visual assessment of the larynx (performed by a physician) and the APJ (performed by a speech-language pathologist), contributing to the evaluation of the discriminative capacity of acoustic measures in distinguishing between individuals with and without vocal disorders.

In clinical practice, different types of tasks can be used to identify vocal disorders. The task selected for acoustic analysis influences its outcome. The vowel [a] is widely used in acoustic and spectrographic vocal analysis due to its stable production and representativeness of vocal resonance. Recent studies indicate that the choice of vowel can significantly influence diagnostic accuracy, with [a] being the most internationally adopted because it facilitates the standardization of procedures and the comparison of results^(12)^. The predominance of the vowel [a] in acoustic protocols is associated with the ease of obtaining sustained speech samples with minimal articulatory interference, allowing for detailed spectrographic analysis of formants and vocal temporal characteristics^(13)^. However, the literature lacks certain cutoff values, and there is a need to identify and validate acoustic measures with robust diagnostic performance for the sustained vowel [a] task in BP speakers.

The aim of the present study was to define the cutoff values ​​of acoustic measures extracted from the sustained vowel [a] for discrimination between the presence and absence of vocal disorders in BP speakers.

## METHODS

This is a cross-sectional, retrospective, and descriptive study that followed the guidelines of the Standards for Reporting Diagnostic Accuracy (STARD)^(14)^. The study was approved by the Institutional Research Ethics Committee under the number 02954612.9.0000.5188. All participants signed the Informed Consent Form.

In the study, the index test was the acoustic analysis of the sustained vowel [a], and the gold standard was the presence or absence of a vocal disorder, defined based on a clinical evaluation consisting of a laryngological examination and APJ. Although the index test assessed the voice (acoustic measures), the target condition to be detected was the presence of a vocal disorder. This study used results derived from the agreement between the visual examination of the larynx and the APJ results as the gold standard to determine the clinical outcome: the presence or absence of a vocal disorder.

The study sample was defined based on inclusion and exclusion criteria applied through procedures that ensured the analysis of the reference standard. Adults aged 18 to 65 years who had undergone an otolaryngological evaluation two weeks prior to data collection, with a confirmed diagnosis of vocal disorder, and without prior vocal treatment (therapy or surgery) before data collection were included. Participants excluded were those with cognitive or neurological disorders that prevented the use of recording procedures, professional voice users, and individuals who had already undergone formal vocal therapy or surgery in the head or neck region. In addition, exclusion criteria related to the quality of vocal signals were also applied, excluding voice samples that had a maximum phonation time of less than 5 seconds, the presence of a cutoff peak in the acoustic signal, and a signal-to-noise ratio (SNR) below 30 dB of sound pressure level (SPL)^(11)^.

Since the research aimed to define the cutoff values ​​of the acoustic measures extracted from the sustained vowel [a] task for discrimination between voices with and without vocal disorders in BP speakers, the eligibility criteria allowed for the inclusion of individuals in both groups. The group without vocal disorders consisted of individuals without vocal complaints, structural or functional laryngeal disorders, and without deviations in vocal quality as demonstrated by the APJ. The group with vocal disorders consisted of individuals with vocal complaints, structural or functional laryngeal disorders, and deviations in vocal quality detected by the APJ.

A sample size calculation based on population size (Equation 1) was performed to estimate how many participants were needed for each group. The parameters used were: population size with vocal disorders (N) in Brazil of 15,232,500 (203,100,000 x 0.075)^(15,16)^, margin of error (e) of 10%, confidence level of 95% (z=1.96), and population proportion of individuals with non-teaching vocal disorders (p) of 0.5. A sample size (n) of 97 participants per group was calculated.


$$Sample\;Size\;\left(n\right)=\frac{\frac{z^{2}.\;p\;\left(1-p\right)}{e^{2}}}{1+\left(\;\frac{z^{2}.\;p\;\left(1-p\right)}{e^{2}N}\right)}$$ (1)

Being:

z = 1.96

p = 0.5

e = 0.1

N = 15,232,000 (7.5% of non-teaching individuals with dysphonia are found in a population of 203,100,000 in Brazil)

Initially, 391 individuals were included (304 women and 87 men). Of these, 15 were excluded based on the following criteria: three individuals with an inconclusive diagnosis of voice disorder to avoid inconsistency in the dataset and to maintain the clinical relevance of the study results; 12 individuals were voice professionals. Thus, the study sample consisted of 376 participants: 277 with voice disorder (217 women and 60 men) and 99 without voice disorder (71 women and 28 men), with a mean age of 41.20 ± 14.04 years.

The recruitment of participants with voice disorders was carried out from the waiting list of a university voice laboratory. All patients sought the service spontaneously or were referred by otolaryngologists. Participants without voice disorders were recruited from students and staff of the university where the research was conducted.

The vocal sample collection procedures followed the laboratory's routine initial vocal assessment protocol. All data were recorded digitally (voice signals) or in medical records (anamnesis and laryngeal examination report). Vocal samples were collected during the first clinical vocal assessment, before vocal therapy started. All participants answered a brief medical history interview and were referred for visual laryngeal examination by an otolaryngologist, with a request to return the report within 15 days.

The following equipment was used for recording: Fonoview software (version 4.5, CTS Informática), Dell all-in-one computer, Sennheiser E-835 cardioid unidirectional microphone (on stand), coupled to a Behringer U-Phoria UMC 204 preamplifier. The recordings were conducted in an acoustic booth with background noise below 20 dB SPL, a sampling rate of 44,000 Hz, and 16 bits. The microphone was positioned 10 cm from the speaker's mouth at a 45º angle. The distance was measured with a ruler before each task, and the participant was positioned at the rear edge of the booth to maintain the fixed distance. The participant was instructed not to move their head.

All participants recorded the sustained vowel [a] speech task at their self-selected habitual frequency and intensity. During recording, the signals were visually monitored using Fonoview software, observing vowel duration (minimum of 5 s) and the presence of peak clipping. When necessary, the recording was repeated to ensure its quality. After collection, participants were referred for an otolaryngological medical examination with videolaryngostroboscopy. These examinations were performed in the same laboratory and resulted in a written report.

For the APJ, vocal samples were presented to three speech-language pathologists, with over 20 years of experience in voice therapy. The APJ session took place in a quiet environment, with headphones connected to a laptop, at a comfortable volume, self-reported by the evaluators. For the APJ, the speech-language pathologists listened to the vocal sample and then marked it on a Visual Analogue Scale (VAS) with a range between 0 and 100 mm. The VAS cutoff values^(17)^ were used to classify the voices according to the presence of vocal deviation and the overall degree (G). A score closer to 0 represents less deviation in vocal quality, and closer to 100 more deviant of the vocal quality^(17)^. Voices assessed with values ​​≤35.5 mm were considered without vocal disorder, while voices with values ​​>35.5 mm were classified as having a vocal disorder. At the end of the APJ session, 20% (n=76) of the vocal samples were randomly repeated to analyze intra-rater reliability using Cohen's kappa coefficient. We selected the results with the highest intra-rater reliability, which obtained a kappa coefficient of 0.89, indicating good agreement.

Before extracting the acoustic measurements analyzed in this study, we used the open-source software Praat^(18)^ (Paul Boersma and David Weenink, University of Amsterdam, Netherlands), version 6.2.10, to obtain the SNR of the vocal signals. The average SNR of the samples included in this research was 43.08 ± 8.02 dB SPL. This value is acceptable (SNR > 30 dB SPL) and guarantees the quality of the signals for the extraction of acoustic measurements^(11)^. None of the vocal signals used in this study presented an RSR < 30 dB SPL. The measurements were automatically extracted using a script in the free software Praat version 6.2.10, VoxMore^(19)^. Forty-four acoustic measurements were extracted, including:

- Fundamental frequency statistics (F0) – eight measures: mean (f_o_mean), median (f_o_median), standard deviation (sdF0), first (f_o_q1) and third quartile (f_o_q3), minimum (f_o_min), maximum (f_o_max) and coefficient of variation (f_o_CV);
- Period statistics – three measures: period average (PER), period standard deviation (PSD), and natural logarithm of the period standard deviation (LNPSD);
- Short-term disturbance of F0 – five measures: local jitter, absolute jitter (jitter_ABS_), jitter relative average perturbation (jitter_RAP_), jitter period perturbation quotient (jitter_PPQ5_) and jitter difference of differences in pitch (jitter_ddp_);
- Short-term amplitude perturbation – seven measures: shimmer_local_, shimmer_dB_, shimmer_APQ1_, shimmer_APQ3_, shimmer_APQ5,_ shimmer_APQ11_ and shimmer_DDA_, amplitude variability index (AVI);
- Spectral and cepstral measures – 16 measures: spectral decay, spectral falloff, six variations of spectral noise level (SNL_100-2600Hz_, SNL_100–3000Hz_, SNL_100–5100Hz_, SNL_100–8000Hz_, SNL_2600–5100Hz_ and SNL_5100–8000Hz_), high-frequency noise (Hfno), difference between the amplitude of the first and second harmonic (H1H2), difference between the amplitude of the first formant and the amplitude of the first harmonic (H1A1), difference between the amplitude of the third formant and the amplitude of the first harmonic (H1A3), spectral flatness of residue signal (SFR), standard deviation of the harmonic-to-noise ratio (HNRDP), harmonic-to-noise ratio Dejonckere (HNRD) and harmonic-to-noise ratio (HNR);
- Reverse filtering – one measure: Pitch amplitude (PA);
- Disturbances in waveform – four measures: autocorrelation and three variations of Glottal to Noise Excitation, including GNE_1000Hz_, GNE_2000Hz_ and GNE_3000Hz._

To generate the ROC curve, the area under the ROC curve was calculated based on the best cutoff point for the analyzed acoustic measures of the sustained vowel, and its performance metrics were obtained. The AUC is a diagnostic performance measure of a test and can range from 0.5 to 1; the higher the value, the better the test performance, being classified as: excellent (>0.90), good (0.80-0.90), acceptable (0.70-0.80), poor (0.60-0.70), and no acceptable discrimination capacity (< 0.60)^(20)^.

In addition to AUC, the performance of the analyzed measures encompassed the positive predictive value (+PV), the negative predictive value (-PV), the positive likelihood ratio (+LR), and the negative likelihood ratio (-LR). In the present study, only the results of acoustic measures with accuracy, sensitivity, and/or specificity values ​​≥ 0.70, considered satisfactory in this research, will be presented. Values ​​less than 0.70 do not have acceptable discriminatory capacity to detect voices with and without vocal disorders^20.^ Thus, cutoff points and diagnostic accuracy indices (AUC, sensitivity, specificity, +LR, −LR, +PV, and −PV) were defined for only 23 of the 44 extracted and analyzed acoustic measures.

All analyses were performed using the Statistical Package for the Social Sciences (SPSS) software, version 2.0. The significance level adopted was 5%.

## RESULTS

**Figure 1:**
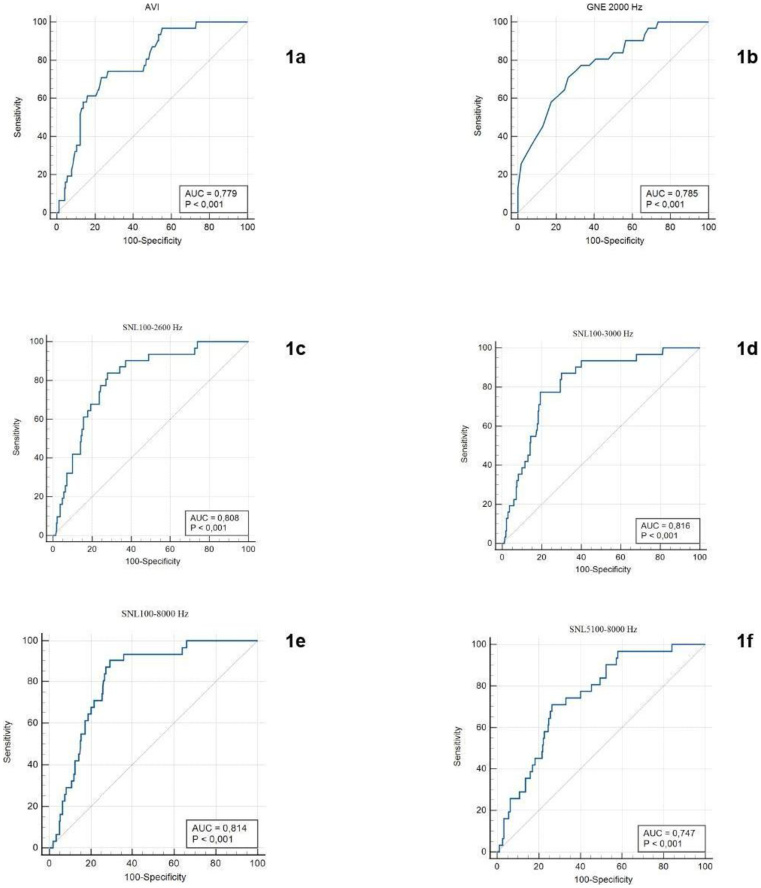
ROC curves of acoustic measures AVI (1a), GNE_2000Hz_ (1b), SNL_100-2600Hz_ (1c), SNL_100-3000Hz_ (1d), SNL_100-8000Hz_ (1e), and SNL_5100-8000Hz_ (1f) from sustained vowel [a] task

Table 1 was subdivided into three sections. It contains the cutoff values ​​and performance metrics of the 23 acoustic measures that presented an AUC above 0.7, among the 44 analyzed in the study. Six acoustic measures showed satisfactory performance, with AUC >0.70, balanced sensitivity and specificity, and values ​​greater than 70%: AVI (≤0.91), GNE_2000Hz_ (>0.92), SNL_100-2600Hz_ (≤7.71), SNL_100-3000Hz_ (≤3.38), SNL_100-8000Hz_ (≤10.95), and SNL_5100-8000Hz_ (≤–1.84) (Figure 1). Ten measures showed satisfactory sensitivity (>70%): GNE_1000Hz_ (>0.92), HNR (>20.73), LNPSD (≤–9.58), PA (>0.91), PSD (≤0.00), shimmer_APQ5_ (≤2.26), shimmer_APQ11_ (≤2.78), shimmer_dB_ (≤0.24), shimmer_local_ (≤2.78), and SNL_100–5100Hz_ (≤11.44). Seven measures showed satisfactory specificity (>70%): GNE_3000Hz_ (>0.9), HNRD (>29.78), Jitter_local_ (≤0.31), Jitter_PPQ5_ (≤0.18), Shimmer_APQ3_ (≤1.18), Shimmer_DDA_(≤3.54), and SNL_2600-5100Hz_ (≤1.21).

**Table 1:** Cutoff point of the acoustic measurements extracted from the sustained vowel [**a**]

**Acoustic Measures**	**Cutoff**	**AUC**	**Sensitivity (%)**	**Specificity(%)**	**+LR**	**-LR**	**+PV**	**-PV**
**AVI**	≤ 0.91	0.779	70.97	76.49	3.02	0.38	25.9	95.8
**GNE_2000 Hz_**	>0.92	0.785	70.97	73.61	2.69	0.39	23.7	95.7
**SNL_100-2600 Hz_**	≤7.71	0.808	83.87	72.12	3.01	0.22	25.7	97.5
**SNL_100-3000 Hz_**	≤3.38	0.816	77.42	80.67	4	0.28	31.6	96.9
**SNL_100-8000 Hz_**	≤10.95	0.814	90.32	70.63	3.08	0.14	26.2	98.4
**SNL_5100-8000 Hz_**	≤-1.84	0.747	70.97	73.61	2.69	0.39	23.7	95.7
**GNE_1000Hz_**	>0.92	0.76	83.87	54.65	1.85	0.3	17.6	96.7
**HNR**	>20.73	0.837	90.32	64.68	2.56	0.15	22.8	98.3
**LNPSD**	≤-9.58	0.722	93.55	48.88	1.83	0.13	17.5	98.5
**PA**	>0.91	0.709	70.97	61.34	1.84	0.47	17.5	94.8
**PSD**	≤0.00	0.722	93.55	48.88	1.83	0.13	17.5	98.5
**SHIMMER_APQ 5_**	≤2.26	0.779	93.55	51.12	1.91	0.13	18.1	98.6
**SHIMMER_APQ 11_**	≤2.78	0.792	100	51.31	2.05	0	19.3	100
**SHIMMER_DB_**	≤0.24	0.78	77.42	66.04	2.28	0.34	20.9	96.2
**SHIMMER_local_**	≤2.78	0.776	77.42	65.67	2.26	0.34	20.7	96.2
**SNL_100-5100 Hz_**	≤11.44	0.807	90.32	69.52	2.96	0.14	25.5	98.4
**GNE_3000 Hz_**	>0.9	0.76	64.52	82.16	3.62	0.43	29.4	95.3
**HNR D**	>29.78	0.72	61.29	76.21	2.58	0.51	22.9	94.5
**JITTER_local_**	≤0.31	0.712	67.74	70.9	2.33	0.46	21.2	95
**JITTER_PPQ5_**	≤0.18	0.725	67.74	70.9	2.33	0.46	21.2	95
**SHIMMER_APQ 3_**	≤1.18	0.765	64.52	74.63	2.54	0.48	22.7	94.8
**SHIMMER_DDA_**	≤3.54	0.765	64.52	75	2.58	0.47	23	94.8
**SNL_2600-5100 HZ_**	≤1.21	0.743	54.84	83.27	3.28	0.54	27.4	94.1

**Caption:** Cutoff: Cutoff value for the acoustic measure.. AUC - Area Under the Curve. +LR: Positive likelihood ratio. Negative likelihood ratio. +PV: Positive predictive value. -PV: Negative predictive value. AVI – Amplitude Variability Index. GNE - Glottal-to-Noise Excitation - GNE_1000Hz_, GNE_2000Hz_, GNE_3000Hz._ SNL - Spectral Noise Level - SNL_100-2600Hz_, SNL_100–3000Hz_, SNL_100–5100Hz,_ SNL_100–8000Hz_, SNL_2600–5100Hz_, SNL_5100–8000Hz._ HNR – Harmonic-to-noise-ratio. LNPSD – Natural logarithm of the standard deviation of the period. PA – Pitch amplitude. PSD – Period standard deviation. Shimmer_APQ5,_ shimmer_APQ11,_ shimmer_dB_, shimmer_local_, shimmer_APQ3_, shimmer_DDA_. HNR D – Harmonic-to-noise ratio Dejonckere. Local Jitter, Jitter_PPQ5 -_ Jitter period perturbation quotient.

## DISCUSSION

This study aimed to define the cutoff values ​​of acoustic measures extracted from the sustained vowel [a] task for discrimination between the presence and absence of vocal disorders in BP speakers. Six acoustic measures obtained good performance in discriminating voices with and without vocal disorders, in addition to specific measures based on sensitivity or specificity, and their cutoff values ​​can be used in clinical vocal practice.

Good accuracy is a combination that reflects the necessary balance between the precise detection of positive cases and the minimization of incorrect diagnoses, being fundamental for the reliable clinical applicability of the evaluated acoustic measures. A measure with high sensitivity has the capacity to correctly identify individuals with vocal disorders, the true positives, being a valuable instrument for screening for vocal disorders, as it minimizes the risk of not detecting true cases. High specificity indicates that the measures are effective in correctly identifying individuals who do not have vocal disorders, the negatives, and can be applied in diagnostic confirmation to avoid unnecessary treatment of individuals without vocal disorders^(4)^.

AVI measure is described in literature as having high sensitivity for predicting the presence of roughness^(21)^. In our research, AVI presented an AUC of 0.779, with a sensitivity of 70.97% and a specificity of 76.49%, suggesting acceptable performance (0.70-0.80) in distinguishing between groups with and without vocal disorders, and may have potential for use as a screening and evaluation tool. Although the +PV of this measure is low (25.9%), the -PV of 95.8% indicates that a negative result can be reliably used to exclude vocal disorders^(22)^, however, a joint analysis with complementary measures is necessary for better decision-making security at the time of evaluation^(23)^.

The measure GNE has proven relevant for voice acoustic analysis by estimating the relative amount of noise in sound signal, reflecting the efficiency of the glottal source compared to the turbulence of the vocal tract^(3)^. GNE can be useful in detecting vocal disorders, especially when combined with other acoustic measures^(24)^. Among its variants, the GNE_2000Hz_ stands out in this study for presenting a more robust balance between diagnostic sensitivity and specificity. GNE_2000Hz_ achieved an AUC of 0.76, with a specificity of 73.61%, demonstrating better performance in correctly identifying individuals without vocal disorders. Clinically, the GNE_2000Hz_ has potential application in differentiating between efficient glottal vocal production and sound signals with a higher noise component, being particularly useful in detecting breathiness and glottal incompetence. Studies with GNE_2000Hz_ reinforce its applicability as a complementary index for vocal disorders detection, especially in multiparametric assessment protocols^(25)^. Regarding the other variations of this measure, despite GNE_1000Hz_ having high sensitivity (83.87%), it demonstrates low specificity (54.65%), resulting in a higher rate of false positives. Despite GNE_3000Hz_ having high specificity (82.16%), it did not demonstrate an advantage in terms of sensitivity and diagnostic stability, which favors the clinical use of GNE_2000Hz_ as a more efficient acoustic measurement.

The acoustic measurement SNL aims to quantify the proportion of aperiodic energy (noise) present in vocal signal^(21)^. The higher the SNL value, the greater the roughness degree^(8)^. Among the performed measures, SNL_100-3000Hz_ presented the highest AUC (0.816), combining a sensitivity of 77.42% and a specificity of 80.67%, providing a good balance for detecting individuals with vocal disorders, making it a viable option in clinical practice^(26)^. Furthermore, it presented the highest +LR (4.00), suggesting that a positive result considerably increases the probability of a vocal disorder^(27)^. SNL_100-8000Hz_ showed one of the highest sensitivities (90.32%), an AUC of 0.814, and excellent -PV, indicating that this measure has strong potential for detecting voice disorders individuals. SNL_100-2600HZ_ presented an AUC of 0.808, with a sensitivity of 83.87% and a specificity of 72.12%. Its -LR was 0.22, indicating that a negative result significantly reduces the probability of vocal disorder presence. The SNL_5100-8000Hz_ measure obtained an AUC of 0.747, with balanced specificity and sensitivity.

Among the measures analyzed, ten performed well in identifying individuals with vocal disorders, but failed to identify individuals without vocal disorders, indicating that they have greater applicability in high-risk populations^(3)^. These measures exhibited high sensitivity, and an AUC greater than 0.7, but low specificity. These are: GNE_1000Hz_, HNR, LNPSD, PA, PSD, shimmer_APQ5_, shimmer_APQ11_, shimmer_dB_, shimmer_local_, and SNL_100-5100HZ_.

Seven other measures performed well in identifying individuals without vocal disorders, but failed to identify individuals with vocal disorders. These measures are applicable in low-risk populations^(3)^, as they showed high specificity and AUC greater than 0.7, but low sensitivity. They are: GNE_3000Hz_, HNRD, jitter_local_, jitter_PPQ5_,, shimmer_APQ3,_ shimmer_DDA_ and SNL_2600–5100Hz_.

HNR parameter stands out as a measure that quantifies the proportion of noise relative to the proportion of harmonics in a vocal sample and can be of great value in differentiating between voices with and without vocal disorders^(28)^. In the present study, the HNR measure obtained the highest AUC among those analyzed (0.837), in addition to high sensitivity (90.32%), suggesting that it is one of the best metrics for detecting the presence of vocal alterations, despite its moderate specificity (64.68%), which indicates a possible number of false positives. This phenomenon is common in highly sensitive tests and should be considered when choosing the best diagnostic approach^(27)^. This finding suggests that HNR may be a promising measure for initial screenings where the priority is to correctly identify positive cases^(29)^. Combining highly sensitive measurements (such as HNR) with others of high specificity (such as SNL_2600-5100Hz_) can optimize diagnostic accuracy. The results highlight the importance of acoustic analysis in voice diagnosis and monitoring, emphasizing the need for a multimodal approach, combining different parameters to increase diagnostic accuracy^(1,25)^.

The definition of cutoff points for acoustic measurements represents an important advance for clinical practice by providing objective parameters that can assist in screening and differential diagnosis between voices with and without vocal disorders. However, it is crucial to emphasize that the lack of satisfactory diagnostic performance for some measurements does not imply their clinical inapplicability. Many of these measurements can be important in specific contexts, such as intra-subject comparison, allowing the monitoring of vocal changes over time, evaluating responses to therapeutic interventions or vocal techniques, and identifying subtle variations that are not captured by purely categorical assessments. Therefore, the integration of multiple acoustic parameters, associated with other procedures of multidimensional voice assessment, favors a more comprehensive and personalized approach to voice management, valuing both robust diagnostic indicators and data that contribute to the continuous monitoring and improvement of vocal health^(30)^.

Every scientific study, by its nature, operates under certain conditions and methodological choices that generate limitations. The present work is no exception, and the considerations that follow aim to offer a transparent analysis of these aspects, outlining the scope and boundaries of applicability of the results. However, although these limitations inform the generalization and context of the findings, they do not compromise the internal validity of the study or the robustness of its main conclusions, which remain relevant and scientifically valid within the population and methodological universe investigated.

The cross-sectional and descriptive design prevents the establishment of direct causal relationships or the evaluation of the stability of the cutoff points over time. Additionally, despite the significant sample size, the imbalance between the number of participants with and without vocal disorders, although reflecting clinical reality, makes it difficult to generalize the cutoff points. Therefore, the cutoff points of this study can be used as a reference in clinical practice, but studies with more comprehensive samples and populations with different prevalences of vocal disorders are necessary. The lack of a recognized gold standard for this purpose can be considered a limitation of the study. The classification of participants as having or not having vocal disorders was based on a combination of visual laryngeal examination, performed by a physician, and the APJ performed by speech-language pathologists. Although the high reliability of the APJ was ensured, its subjective nature may influence categorization. Taken together, these considerations delimit the scope of applicability of the results and highlight the complexity of vocal assessment, while simultaneously expanding and refining knowledge in this area.

As a clinical recommendation, the preferred set of measures in cases of suspected vocal disorder, derived from the sustained vowel [a] task in BP speakers, are: SNL_100–2600Hz,_ SNL_100–3000Hz_, and SNL_100–8000Hz_, since they exhibit sensitivity and specificity ≥80%. For screening purposes, the recommended measures, based on sensitivity ≥80%, are: GNE_1000Hz_, HNR, LNPSD, PSD, Shimmer_APQ5_, Shimmer_APQ11_, and SNL_100–5100Hz._ Finally, for diagnostic confirmation, the measures with specificity of ≥80% include GNE_3000Hz_, and SNL_2600–5100Hz_.

## CONCLUSION

In the present study, out of the 44 acoustic measures extracted from the sustained vowel [a] in BP speakers, 23 presented satisfactory performance metrics. The acoustic measures AVI, GNE_2000Hz_, SNL_100-2600Hz_, SNL_100-3000Hz_, SNL_100-8000Hz_, and SNL_5100-8000Hz_ demonstrated acceptable discriminatory ability to differentiate between voices with and without vocal disorders. The measures GNE_1000Hz_, HNR, LNPSD, PA, PSD, shimmer_APQ5,_ shimmer_APQ11,_ shimmer_dB_, shimmer_local_, and SNL_100–5100Hz_ presented a cutoff point with satisfactory sensitivity and can be used in clinical practice for screening purposes. The measures GNE_3000Hz,_ HNRD, Jitter_local,_ Jitter_PPQ5,_ shimmer_APQ3,_ shimmer_DDA_, and SNL_2600-5100Hz_ presented a cutoff point with satisfactory specificity and are applicable for diagnostic confirmation.
