# Supplementary material for: Accuracy and cut-off values of different acoustic measures in the sustained vowel task of Brazilian Portuguese speakers
Source: Codas. 2026 Jul 3;38(4):e20250288. doi: 10.1590/2317-1782/e20250288en (PMC13330937; doi:10.1590/2317-1782/e20250288en)
Supplement: Translation

# Translation: Acurácia e ponto de corte de diferentes medidas acústicas na tarefa de vogal sustentada em falantes do português brasileiro

**DOI:** 10.1590/2317-1782/e20250288pt

**Authors:** Amanda Feliciano, Mara Behlau, Jordana Balbinot, Samuel Ribeiro de Abreu, Vanessa Veis Ribeiro, Leonardo Wanderley Lopes

**Affiliations:** 1 Centro de Estudos da Voz – CEV - São Paulo (SP), Brasil.; 2 Universidade Federal de Rondonópolis – UFR - Rondonópolis (MS), Brasil.; 3 Departamento de Fonoaudiologia, Faculdade de Ciências e Tecnologias em Saúde, Universidade de Brasília – UnB - Brasília (DF), Brasil.; 4 Departamento de Fonoaudiologia, Centro de Ciências da Saúde, Universidade Federal da Paraíba – UFPB - João Pessoa (PB), Brasil.

**Keywords:** Voz, Acústica, Distúrbios da Voz, Disfonia, Diagnóstico

## Abstract

**Objetivo:**

Definir os valores de corte das medidas acústicas extraídas da tarefa de vogal sustentada [a] para discriminação entre presença e ausência de distúrbio vocal em falantes do português brasileiro.

**Método:**

A amostra foi composta por 376 participantes, 288 do gênero feminino e 88 do gênero masculino, com idade média de 41,20 ± 14,04 anos. Os indivíduos foram submetidos ao exame de avaliação laringológica, e a uma gravação vocal, sendo coletada uma amostra de fala com a vogal [a] sustentada e posteriormente realizado o julgamento perceptivo auditivo e acústico da voz, dos quais 277 foram classificados com distúrbio vocal e 99 sem distúrbio vocal. Foram extraídas 44 medidas acústicas e analisadas por meio da curva ROC para identificação do ponto de corte de cada medida, bem como suas métricas de desempenho.

**Resultados:**

Foram definidos pontos de corte para 23 das 44 medidas acústicas inicialmente extraídas. Dentre elas, seis medidas demonstraram desempenho robusto e clinicamente relevante para discriminar vozes com e sem distúrbio vocal em falantes do português brasileiro. As medidas com valores de AUC superiores a 0,70 e sensibilidade e especificidade equilibradas acima de 70% foram: Amplitude Variability Index (AVI) (≤0,91), Glottal-to-noise excitation (GNE)2000Hz (>0,92), Spectral Noise Level (SNL)100-2600Hz (≤7,71), SNL100-3000Hz (≤3,38), SNL100-8000Hz (≤10,95) e SNL5100-8000Hz (≤–1,84).

**Conclusão:**

Seis medidas apresentaram desempenho satisfatório para identificar a presença de distúrbio vocal em falantes do português brasileiro. Dez medidas apresentaram bom desempenho para triagem vocal e sete para confirmação diagnóstica de distúrbio vocal.

## INTRODUÇÃO

A análise acústica faz parte dos procedimentos de avaliação multidimensional da voz em conjunto com o julgamento perceptivo auditivo (JPA), a análise aerodinâmica, o exame estrutural e vibratório da laringe, e a autoavaliação vocal^(1)^. A partir da análise acústica é possível obter medidas para quantificar as características do sinal vocal e realizar a avaliação descritiva dos padrões visuais desses sinais^(2)^. Isso contribui para triagem, confirmação diagnóstica e para o monitoramento da eficácia das intervenções terapêuticas em sujeitos com distúrbio vocal^(3,4)^.

Há uma grande diversidade de medidas acústicas para avaliação do sinal vocal. Dentre as medidas convencionais e mais amplamente utilizadas estão a frequência fundamental ou de oscilação (f_o_), medidas de curto termo de perturbação de frequência (*jitter*), de intensidade vocal (*shimmer*) e Proporção Harmônico Ruído (*Harmonic-Noise Ratio* - HNR)^(1)^. As medidas convencionais são eficazes, sobretudo, em casos de desvios vocais leves. Já as medidas espectrais e cepstrais, que vem sendo mais amplamente utilizadas e estudadas nos últimos anos, resultam da dupla transformação do sinal acústico da voz, que revelam o grau de organização dos harmônicos no espectro, sendo efetivas para análise de vozes desviadas, visto que são fortes preditoras da presença de desvio vocal^(5)^.

A aplicabilidade clínica das medidas acústicas depende da definição de valores de corte conhecidos e válidos, com desempenho diagnóstico consistente e adequado à população-alvo. No contexto do português brasileiro (PB), poucas medidas acústicas têm valores de corte definidos, o que limita a comparação intersujeitos e a comparação da voz do paciente com valores de referência^(6)^, não contribuindo para uma prática clínica embasada e consciente.

Recentemente, foram estabelecidos valores de corte para dois índices multiparamétricos, o *Acoustic Voice Quality Index* (AVQI) e o *Acoustic Breathiness Index* (ABI) para auxiliar na identificação de alterações vocais em falantes do PB^(7)^. A acurácia diagnóstica do AVQI foi excelente e o ponto de corte foi de 1,33, apresentando sensibilidade de 78,8% e especificidade de 90,6%. Já o ABI apresentou acurácia diagnóstica boa, com ponto de corte de 2,94, sensibilidade de 75,3% e especificidade de 93,4%^(7)^. Esses dados evidenciam a robustez do AVQI e do ABI como ferramentas objetivas para a avaliação e monitoramento de distúrbios vocais^(7,8)^.

Outras duas medidas têm ganhado destaque como parâmetros objetivos na análise acústica da voz, o *Cepstral Peak Prominence* (CPP) e o *Smoothed Cepstral Peak Prominence* (CPPS), visto que apresentam boa correlação com o julgamento perceptivo-auditivo e alta sensibilidade na detecção de desvios vocais e não dependem da extração da frequência fundamental. Há valores de corte para discriminar vozes com e sem distúrbio vocal no PB^(6)^. O estudo identificou que valores abaixo de 28,15 decibéis (dB) (vogal [ɛ]), 28,77 dB (vogal [a]) e 28,58 dB (frase *Consensus Auditory-Perceptual Evaluation of Voice* - CAPE‑V) e para o CPPS valores abaixo de 16,42 dB (vogal [ɛ]), 17,02 dB (vogal [a]) e 11,30 dB (todas as sentenças do CAPE-V) são indicativos de presença de distúrbio vocal^(6)^.

O ponto de corte é extraído a partir da melhor relação entre sensibilidade e especificidade. Nessa análise, é essencial a identificação de medidas com desempenho equilibrado, que apresentam simultaneamente valores satisfatórios de sensibilidade e especificidade. Essas medidas têm a capacidade de identificar corretamente tanto indivíduos com distúrbios vocais (verdadeiros positivos) quanto indivíduos sem a condição (verdadeiros negativos), reduzindo a ocorrência de falsos diagnósticos. Esse equilíbrio é geralmente representado por valores de área sob a curva (AUC - *Area Under the Curve*) superiores a 0,70 em análises ROC, indicando bom desempenho discriminativo^(1)^.

A classificação quanto à presença ou ausência de condições específicas de saúde depende da precisão do procedimento de medição ou teste. Tal precisão reflete a concordância entre os achados de um novo teste, chamado teste índice, e os obtidos por meio do método mais confiável existente para detectar o desfecho de saúde em questão, o padrão de referência^(9)^. Portanto, a seleção de um padrão de referência apropriado é imperativa para validar as métricas de acurácia de um teste diagnóstico emergente. Em relação à escolha do teste de referência, os clínicos enfrentam obstáculos ao estabelecer critérios para normalidade vocal devido ao seu caráter intrinsecamente multidimensional. Atualmente, não existe um único método de referência capaz de diferenciar com precisão indivíduos vocalmente saudáveis daqueles com distúrbio vocal^(10)^. Na ausência de evidências científicas robustas e consenso entre especialistas sobre a detecção de certas condições de saúde, padrões de referência alternativos devem ser desenvolvidos^(9)^.

Embora o JPA e a autoavaliação sejam tradicionalmente utilizadas como métodos preliminares para identificar distúrbios vocais^(11)^, a inspeção visual da laringe (incluindo laringoscopia e estroboscopia) é considerada o padrão-ouro para confirmar a categorização diagnóstica dos distúrbios da voz^(10)^. A síntese de informações de procedimentos distintos para construir um critério de referência integrativo, derivado da correlação entre os resultados de uma avaliação visual da laringe (realizada por um médico) e da JPA (realizada por um fonoaudiólogo), pode servir para avaliar a capacidade discriminativa de medidas acústicas na distinção entre indivíduos com e sem distúrbios vocais.

Na prática clínica da área de voz, pode-se utilizar diferentes tipos de tarefas para a identificação de distúrbios vocais. O tipo de tarefa selecionado para a análise acústica, influencia diretamente no seu resultado. A vogal [a] é amplamente utilizada na análise acústica e espectrográfica vocal devido à sua produção estável e representatividade da ressonância vocal. Estudos recentes indicam que a escolha da vogal pode influenciar significativamente a precisão diagnóstica, sendo [a] a mais adotada internacionalmente por facilitar a padronização dos procedimentos e a comparação entre resultados^(12)^. A predominância da vogal [a] em protocolos acústicos está também associada à facilidade de obtenção de amostras de fala sustentada com mínima interferência articulatória, o que favorece a análise espectrográfica detalhada dos formantes e das características temporais da voz^(13)^. Porém, observa-se na literatura a ausência de alguns valores de corte e a necessidade de identificar e validar medidas acústicas com desempenho diagnóstico robusto para a tarefa da vogal sustentada [a] em falantes do PB.

Nesse contexto, o objetivo do presente estudo foi definir os valores de corte das medidas acústicas extraídas na tarefa de vogal sustentada [a] para discriminação entre presença e ausência de distúrbio vocal em falantes do PB.

## MÉTODO

Trata-se de um estudo transversal, retrospectivo e descritivo. O estudo seguiu as diretrizes do *Standards for Reporting Diagnostic Accuracy* (STARD)^(14)^. O estudo foi aprovado pelo Comitê de Ética em Pesquisa da instituição responsável sob o número 02954612.9.0000.5188. Todos os participantes assinaram o Termo de Consentimento Livre e Esclarecido (TCLE).

No presente estudo o teste índice foi a análise acústica da vogal sustentada [a] e o teste padrão de referência (*gold standard*) foi a presença ou ausência de distúrbio vocal, definida com base na avaliação clínica composta por exame laringológico e JPA. Assim, apesar do teste índice avaliar a voz (medidas acústicas), a condição-alvo que buscou-se detectar/discriminar foi a presença de distúrbio vocal. O presente estudo utilizou um resultado derivado da concordância entre o exame visual da laringe e os resultados da JPA como padrão de referência para determinar o desfecho clínico, ou seja, a presença ou ausência de distúrbio vocal.

A amostra do estudo foi constituída a partir de critérios de inclusão e exclusão aplicados por meio dos procedimentos que garantiram a análise do padrão de referência. Foram incluídos no estudo adultos entre 18 e 65 anos que haviam realizado avaliação otorrinolaringológica nas duas semanas anteriores à coleta dos dados, com diagnóstico confirmado de distúrbio vocal, e que não haviam iniciado tratamento vocal (terapia ou cirurgia) antes da coleta. Foram excluídos participantes com distúrbios cognitivos ou neurológicos que impedissem o uso dos procedimentos de gravação, usuários profissionais da voz, e indivíduos que já haviam feito terapia vocal formal ou cirurgia na região da cabeça ou pescoço. Além disso, também foram aplicados critérios de exclusão relacionados à qualidade dos sinais vocais, excluindo-se amostras de voz que possuíam tempo máximo de fonação menor do que 5 segundos, presença de pico de corte no sinal acústico e relação sinal-ruído (RSR) abaixo de 30 dB de nível de pressão sonora (NPS)^(11)^.

Como o objetivo da pesquisa foi definir os valores de corte das medidas acústicas extraídas na tarefa de vogal sustentada [a] para discriminação entre vozes com e sem distúrbio vocal em falantes do PB, os critérios de elegibilidade permitiram a inclusão de indivíduos em ambos os grupos. O grupo sem distúrbio vocal foi composto por indivíduos sem queixas vocais, distúrbios laríngeos estruturais ou funcionais e sem desvio na qualidade vocal conforme demonstrado pelo JPA. O grupo com distúrbio vocal foi composto por indivíduos com queixas vocais, distúrbio estrutural ou funcional da laringe e desvio na qualidade vocal detectado pelo JPA.

Foi realizado um cálculo do tamanho da amostra pelo tamanho da população (Equação 1) para estimar quantos participantes eram necessários para cada grupo de pesquisa. Os parâmetros utilizados foram: tamanho da população com distúrbio vocal (N) no Brasil de 15.232.500 (203.100.000*0,075)^(15,16)^, margem de erro (e) de 10%, nível de confiança de 95% (z=1,96) e proporção populacional de indivíduos com distúrbio vocal não professores (p) de 0,5. Calculou-se um tamanho amostral (n) de 97 participantes, por grupo.


$$Tamanho\;da\;amostra\;\left(n\right)=\frac{\frac{z^{2}.\;p\;\left(1-p\right)}{e^{2}}}{1+\left(\;\frac{z^{2}.\;p\;\left(1-p\right)}{e^{2}N}\right)}$$ (1)

Sendo:

z = 1,96

p = 0,5

e = 0,1

N = 15.232.000 (7,5% de disfônicos não professores em 203.100.000 habitantes no Brasil)

Inicialmente foram incluídos 391 indivíduos (304 mulheres e 87 homens). Desses, 15 foram excluídos com base nos seguintes critérios: foram excluídos três indivíduos com diagnóstico inconclusivo de distúrbio de voz para evitar inconsistência no conjunto de dados e manter a relevância clínica dos resultados do estudo; 12 indivíduos eram profissionais da voz. Assim, a amostra do estudo foi composta por 376 participantes: 277 pacientes com distúrbio vocal (217 mulheres e 60 homens) e 99 indivíduos sem distúrbio vocal (71 mulheres e 28 homens), com média de idade de 41,20 ± 14,04 anos.

O recrutamento dos participantes com distúrbio vocal foi realizado a partir da lista de espera de um laboratório de voz de uma universidade. Todos os pacientes buscaram o serviço por demanda espontânea ou foram encaminhados por otorrinolaringologistas. Os participantes sem distúrbio vocal foram recrutados entre estudantes e funcionários da universidade onde a pesquisa foi realizada.

Os procedimentos de coleta de amostras vocais seguiram o protocolo rotineiro de avaliação vocal inicial do laboratório. Todos os dados foram registrados digitalmente (sinais de voz) ou em prontuário (anamnese e laudo do exame laríngeo). As amostras vocais foram coletadas durante a primeira avaliação clínica vocal, antes do início da terapia vocal. Todos os participantes responderam a uma breve entrevista de histórico médico e foram encaminhados para exame laríngeo visual por um otorrinolaringologista, com solicitação de retorno do laudo em até 15 dias.

Os seguintes equipamentos foram utilizados para gravação: *software Fonoview* (versão 4.5, CTS Informática), computador Dell *all-in-one*, microfone unidirecional cardioide Sennheiser modelo E-835 (em pedestal), acoplado a um pré-amplificador Behringer U-Phoria UMC 204. As gravações foram feitas em cabine acústica com ruído de fundo inferior a 20 dB SPL, taxa de amostragem de 44.000 Hz e 16 bits. O microfone foi posicionado a 10 cm da boca do falante em um ângulo de 45º. A distância foi medida com régua antes de cada tarefa, e o participante era posicionado no limite traseiro da cabine para manter a distância fixa. Orientou-se que o participante não movimentasse a cabeça.

Todos os participantes registraram a tarefa de fala da vogal sustentada [a], em frequência e intensidade habitual auto selecionada. Durante a gravação, os sinais foram monitorados visualmente no *software Fonoview,* observando a duração das vogais (mínimo de 5 s) e a presença de *peak clipping*. Quando necessário, a gravação foi repetida para garantir a qualidade dos registros. Após a coleta, os participantes foram encaminhados para exame médico otorrinolaringológico com videolaringoestroboscopia. Tais exames foram realizados no mesmo laboratório e resultaram em laudo escrito.

Para o JPA as amostras vocais foram apresentadas a três fonoaudiólogos especialistas em voz, com mais de 20 anos de experiência em voz. A sessão de JPA ocorreu em um ambiente silencioso, com fones de ouvido acoplados a um *laptop,* em volume confortável, autorreferido pelos avaliadores. Para o JPA, os fonoaudiólogos escutaram a amostra vocal e na sequência faziam a marcação em uma Escala Visual Analógica (EVA) com intervalo entre 0 e 100 mm. Os valores de corte da EVA^(17)^ foram utilizados para classificar as vozes quanto à presença de desvio vocal e o grau geral (GG). Uma pontuação mais próxima de 0 representa menor desvio na qualidade vocal, e quanto mais próxima de 100, mais desviante é a qualidade vocal^(17)^. Sinais avaliados com valores ≤ 35,5 mm foram considerados sem distúrbio vocal, enquanto vozes com valores > 35,5 mm foram classificadas com distúrbio vocal. Ao final da sessão de JPA, 20% (n=76) das amostras vocais foram repetidas aleatoriamente para analisar a confiabilidade intra-avaliador usando o coeficiente kappa de Cohen. Selecionamos os resultados com a maior confiabilidade intra-avaliador, que obtiveram um coeficiente kappa de 0,89, indicando boa concordância.

Antes de realizar a extração das medidas acústicas analisadas neste estudo, utilizamos o software de acesso aberto Praat^(18)^ (Paul Boersma e David Weenink, Universidade de Amsterdã, Holanda), versão 6.2.10, para obter a RSR dos sinais vocais. A RSR média das amostras incluídas nesta pesquisa foi de 43,08 ± 8,02 dB NPS. Este valor é aceitável (RSR > 30 dB NPS) e garante a qualidade dos sinais para a extração das medidas acústicas^(11)^. Nenhum dos sinais vocais utilizados neste estudo apresentou RSR < 30 dB NPS. As medidas foram extraídas automaticamente por meio de um script no software gratuito Praat versão 6.2.10, o *VoxMore* ^(19)^. Foram extraídas 44 medidas acústicas, incluindo:

- Estatísticas da frequência fundamental (f_o_) – oito medidas: média (f_o_media), mediana (f_o_mediana), desvio-padrão (DPf_o_) primeiro (f_o_q1) e terceiro quartil (f_o_q3), mínima (f_o_min), máxima (f_o_max) e coeficiente de variação (f_o_CV);
- Estatísticas do período – três medidas: média do período (PER), desvio-padrão do período (PSD) e logaritmo natural do desvio-padrão do período (LNPSD);
- Perturbação de curto termo de f_o_ – cinco medidas_:_ *jitter* local, *jitter* absoluto (*jitter_ABS_* ), *jitter relative average perturbation (jitter*_RAP_), *jitter period perturbation quotient* (*jitter*_PPQ5_), *jitter difference of diferences in pitch* (*jitter*_ddp_);
- Perturbação de curto termo da amplitude – sete medidas: *shimmer*_local_, *shimmer*_dB_, *shimmer*_APQ1_, *shimmer*_APQ3_, *shimmer*_APQ5,_ *shimmer*_APQ11_ e *shimmer*_DDA_, *amplitude variability index* (AVI);
- Medidas espectrais e cepstrais – 16 medidas: declínio espectral, queda espectral, seis variações do *spectral noise level* (SNL_100-2600 Hz_, SNL_100–3000 Hz_, SNL_100–5100 Hz_, SNL_100–8000 Hz_, SNL_2600–5100 Hz_ e SNL_5100–8000 Hz_), *high-frequency noise* (Hfno), diferença entre a amplitude do primeiro e do segundo harmônico (H1H2), diferença entre a amplitude do primeiro formante e a amplitude do primeiro harmônico (H1A1), diferença entre a amplitude do terceiro formante e a amplitude do primeiro harmônico (H1A3), *spectral flatness of residue signal* (SFR), desvio-padrão do *harmonic-to-noise ratio* (HNRDP), *harmonic-to-noise ratio* Dejonckere (HNRD) e *harmonic-to-noise-ratio* (HNR).
- Filtragem inversa – uma medida: *Pitch amplitud* (PA).
- Perturbações na forma da onda – quatro medidas: autocorrelação e três variações do *Glottal to Noise Excitation*, incluindo GNE_1000Hz_, GNE_2000Hz_ e GNE_3000Hz._

Para gerar a curva ROC foi calculada a área sob a curva ROC a partir do melhor ponto de corte para as medidas acústicas analisadas da vogal sustentada e obteve-se suas métricas de desempenho. A AUC trata-se de uma medida de desempenho diagnóstico de um teste e pode variar de 0,5 a 1, quanto mais alta, melhor o desempenho do teste, sendo classificada como: excelente (> 0,90), boa (0,80-0,90), aceitável (0,70-0,80), ruim (0,60-0,70) e nenhuma capacidade de discriminação aceitável (< 0,60)^(20)^.

Além da AUC, o desempenho das medidas analisadas levou em consideração o valor preditivo positivo (+PV), o valor preditivo negativo (-PV), a razão de verossimilhança positiva (+LR) e a razão de verossimilhança negativa (-LR). No presente estudo, serão apresentados apenas os resultados das medidas acústicas com valores de acurácia, sensibilidade e/ou especificidade ≥ 0,70, considerados na presente pesquisa como satisfatórios. Valores menores que 0,70 são considerados sem capacidade discriminatória aceitável para detectar vozes com e sem distúrbio vocal^(20)^. Assim, das 44 medidas extraídas e analisadas, foi possível definir ponto de corte e índices de acurácia diagnóstica - AUC, sensibilidade, especificidade, +LR, -LR, +PV e -PV de apenas 23 medidas acústicas.

Todas as análises foram realizadas utilizando o software *Statistical Package for the Social Sciences* (SPSS), versão 2.0. O nível de significância adotado foi de 5%.

## RESULTADOS

A Tabela 1 foi subdividida em três sessões onde podem ser encontrados os valores de corte e as métricas de desempenho das 23 medidas acústicas que apresentaram AUC acima de 0,7, dentre as 44 analisadas. Foram definidos os seguintes pontos de corte: seis medidas acústicas apresentaram desempenho satisfatório com AUC >0,70, sensibilidade e especificidade equilibradas e superiores a 70%: AVI (≤0,91), GNE_2000Hz_ (>0,92), SNL_100-2600Hz_ (≤7,71), SNL_100-3000Hz_ (≤3,38), SNL_100-8000Hz_ (≤10,95) e SNL_5100-8000Hz_ (≤–1,84) (Figura 1). Dez medidas apresentaram sensibilidade satisfatória (>70%), foram elas: GNE_1000Hz_ (>0,92), HNR (>20,73), LNPSD (≤–9,58), PA (>0,91), PSD (≤0,00), *shimmer*_APQ5_ (≤2,26), *shimmer*_APQ11_ (≤2,78), *shimmer*_dB_ (≤0,24), *shimmer*_local_ (≤2,78) e SNL_100–5100Hz_ (≤11,44). Já sete apresentaram especificidade satisfatória (>70%): GNE_3000Hz_ (>0,9), HNRD (>29,78), Jitter_local_ (≤0,31), Jitter_PPQ5_ (≤0,18), Shimmer_APQ3_ (≤1,18), Shimmer_DDA_(≤3,54) e SNL_2600-5100Hz_ (≤1,21).

**Tabela 1:** Ponto de corte das medidas acústicas extraídas a partir da vogal [a] sustentada

**Medidas Acústicas**	***CUT-OFF***	**AUC**	**Sensibilidade (%)**	**Especificidade (%)**	**+LR**	**-LR**	**+PV**	**-PV**
**AVI**	≤ 0.91	0.779	70,97	76.49	3.02	0.38	25.9	95,8
**GNE_2000 HZ_**	>0,92	0,785	70,97	73,61	2,69	0,39	23,7	95,7
**SNL_100-2600 HZ_**	≤7,71	0,808	83,87	72,12	3,01	0,22	25,7	97,5
**SNL_100-3000 HZ_**	≤3,38	0,816	77,42	80,67	4	0,28	31,6	96,9
**SNL_100-8000 HZ_**	≤10,95	0,814	90,32	70,63	3,08	0,14	26,2	98,4
**SNL_5100-8000 HZ_**	≤-1,84	0,747	70,97	73,61	2,69	0,39	23,7	95,7
**GNE_1000HZ_**	>0,92	0,76	83,87	54,65	1,85	0,3	17,6	96,7
**HNR**	>20,73	0,837	90,32	64,68	2,56	0,15	22,8	98,3
**LNPSD**	≤-9,58	0,722	93,55	48,88	1,83	0,13	17,5	98,5
**PA**	>0,91	0,709	70,97	61,34	1,84	0,47	17,5	94,8
**PSD**	≤0,00	0,722	93,55	48,88	1,83	0,13	17,5	98,5
**SHIMMER_APQ 5_**	≤2,26	0,779	93,55	51,12	1,91	0,13	18,1	98,6
**SHIMMER_APQ 11_**	≤2,78	0,792	100	51,31	2,05	0	19,3	100
**SHIMMER_DB_**	≤0,24	0,78	77,42	66,04	2,28	0,34	20,9	96,2
**SHIMMER LOCAL**	≤2,78	0,776	77,42	65,67	2,26	0,34	20,7	96,2
**SNL_100-5100 HZ_**	≤11,44	0,807	90,32	69,52	2,96	0,14	25,5	98,4
**GNE_3000 HZ_**	>0,9	0,76	64,52	82,16	3,62	0,43	29,4	95,3
**HNR D**	>29,78	0,72	61,29	76,21	2,58	0,51	22,9	94,5
**JITTER LOCAL**	≤0,31	0,712	67,74	70,9	2,33	0,46	21,2	95
**JITTER_PPQ5_**	≤0,18	0,725	67,74	70,9	2,33	0,46	21,2	95
**SHIMMER_APQ 3_**	≤1,18	0,765	64,52	74,63	2,54	0,48	22,7	94,8
**SHIMMER_DDA_**	≤3,54	0,765	64,52	75	2,58	0,47	23	94,8
**SNL_2600-5100 HZ_**	≤1,21	0,743	54,84	83,27	3,28	0,54	27,4	94,1

**Legenda:***CUT-OFF*: Valor de corte para a medida acústica. AUC (*Area Under the Curve*): Área sob a curva. +LR: Razão de verossimilhança positiva. -LR: Razão de verossimilhança negativa. +PV: Valor preditivo positivo. -PV: Valor preditivo negativo. AVI – *Amplitude Variability Index.* GNE - *Glottal-to-Noise Excitation* - GNE_1000Hz_, GNE_2000Hz_, GNE_3000Hz._ SNL *(Spectral Noise Level)* - SNL_100-2600 Hz_, SNL_100–3000 Hz_, SNL_100–5100 Hz,_ SNL_100–8000 Hz_, SNL_2600–5100 Hz_, SNL_5100–8000 Hz._ HNR – *Harmonic-to-noise-ratio.* LNPSD – Logaritmo natural do desvio-padrão do período. PA – *Pitch amplitud*. PSD – *Desvio-padrão do período. Shimmer*_APQ5,_ *shimmer*_APQ11,_ *shimmer*_dB_, *shimmer*_local_, *shimmer*_APQ3_, *shimmer*_DDA_. HNR D – *Harmonic-to-noise ratio* Dejonckere. J*itter* local, *Jitter*_PPQ5 -_ *jitter period perturbation quotient*.

**Figura 1:** Curvas ROC das medidas acústicas AVI (1a), GNE_2000Hz_ (1b), SNL_100-2600Hz_ (1c), SNL_100-3000Hz_ (1d), SNL_100-8000Hz_ (1e) e SNL_5100-8000Hz_ (1f) na tarefa da vogal [a] sustentada

## DISCUSSÃO

O presente estudo se propôs a definir os valores de corte das medidas acústicas extraídas na tarefa de vogal sustentada [a] para discriminação entre vozes com e sem distúrbio vocal em falantes do PB. No presente estudo, seis medidas acústicas apresentaram bom desempenho para discriminar vozes com e sem distúrbio vocal, além de medidas específicas com base em sensibilidade ou especificidade, e seus valores de corte podem ser utilizados na prática clínica vocal.

Uma boa acurácia é uma combinação que reflete o equilíbrio necessário entre a detecção precisa de casos positivos e a minimização de diagnósticos incorretos, sendo fundamental para a aplicabilidade clínica confiável das medidas acústicas avaliadas. Uma medida com alta sensibilidade possui capacidade de identificar corretamente indivíduos com distúrbio vocal, ou seja, os positivos, sendo um instrumento valioso para a triagem de distúrbio vocal, pois minimiza o risco de não detectar casos verdadeiros. Já a alta especificidade indica que as medidas são eficazes na identificação correta dos indivíduos que não apresentam distúrbio vocal, ou seja, os negativos, sendo aplicadas na confirmação diagnóstica para evitar o tratamento desnecessário de indivíduos sem distúrbios vocais^(4)^.

A medida AVI é descrita na literatura com alta sensibilidade para predizer a presença da rugosidade^(21)^. Em nossa pesquisa, ela apresentou uma AUC de 0,779, com sensibilidade de 70,97% e especificidade de 76,49%, sugerindo um desempenho aceitável (0,70-0,80) na distinção entre grupos com e sem distúrbio vocal, podendo apresentar potencial para ser utilizada como uma ferramenta para triagem e avaliação. Embora o +PV dessa medida seja baixo (25,9%), o -PV de 95,8% indica que um resultado negativo pode ser confiável para excluir o distúrbio vocal^(22)^. No entanto, é necessária uma análise conjunta com medidas complementares para melhor segurança de tomada de decisão no momento da avaliação^(23)^.

A medida acústica GNE tem se mostrado relevante na análise acústica da voz por estimar a quantidade relativa de ruído no sinal sonoro, refletindo a eficiência da fonte glótica em comparação à turbulência do trato vocal^(3)^. O GNE pode ser útil na detecção de distúrbios vocais, especialmente quando combinado com outras medidas acústicas^(24)^. Dentre suas variantes, o GNE_2000Hz_ destaca-se no presente estudo por apresentar um equilíbrio mais robusto entre sensibilidade e especificidade diagnóstica. O GNE_2000Hz_ alcançou AUC de 0,76, com especificidade de 73,61%, evidenciando melhor desempenho na correta identificação de indivíduos sem distúrbio vocal. Clinicamente, o GNE_2000Hz_ tem aplicação potencial na diferenciação entre produção vocal glótica eficiente e sinais sonoros com maior componente de ruído, sendo particularmente útil na detecção de soprosidade e incompetência glótica. Estudos com o GNE_2000Hz_ reforçam sua aplicabilidade como índice complementar na detecção de distúrbios vocais, sobretudo em protocolos multiparamétricos de avaliação^(25)^. No que tange às demais variantes dessa medida, o GNE_1000Hz_, apesar de sua alta sensibilidade (83,87%), demonstra baixa especificidade (54,65%), resultando em maior taxa de falsos positivos. Já o GNE_3000Hz_, apesar de ter alta especificidade (82,16%), seu desempenho geral não demonstrou vantagem em termos de sensibilidade e estabilidade diagnóstica, o que favorece o uso clínico do GNE_2000Hz_ como uma medida acústica mais eficiente.

A medida acústica SNL tem a finalidade de quantificar a proporção de energia aperiódica (ruído) presente no sinal vocal^(21)^. Quanto maior o valor de SNL, maior o grau de rugosidade^(8)^. Dentre as medidas realizadas, a medida SNL_100-3000Hz_ apresentou a maior AUC (0,816), combinando uma sensibilidade de 77,42% e especificidade de 80,67%, fornecendo um bom equilíbrio para detectar indivíduos com distúrbio vocal, tornando-se uma opção viável na prática clínica^(26)^. Além disso, apresentou o maior +LR (4,00), sugerindo que um resultado positivo aumenta consideravelmente a probabilidade de distúrbio vocal^(27)^. A medida SNL_100-8000Hz_ apresentou uma das maiores sensibilidades (90,32%), um AUC de 0,814 e excelente -PV indicando que essa medida tem um forte potencial para detectar indivíduos com distúrbio vocal. A medida SNL_100-260OHZ_ apresentou um AUC de 0,808, com sensibilidade de 83,87% e especificidade de 72,12%. Seu -LR foi de 0,22, indicando que um resultado negativo reduz significativamente a probabilidade da presença de distúrbio vocal. A medida SNL_5100-8000Hz_ obteve AUC de 0,747, especificidade e sensibilidade equilibradas.

Dentre as medidas analisadas, dez obtiveram bom desempenho para identificar indivíduos com distúrbio vocal, porém falham em identificar indivíduos sem distúrbio vocal. Isso indica que essas medidas possuem maior aplicabilidade em populações de alto risco^(3)^, visto que apresentaram alta sensibilidade e AUC maior que 0.7, porém baixa especificidade. São elas: GNE_1000Hz_, HNR, LNPSD, PA, PSD, *shimmer*_APQ5_, *shimmer*_APQ11_, *shimmer*_dB_, *shimmer*_local_, SNL_100-5100HZ_.

Outras sete medidas obtiveram bom desempenho para identificar indivíduos sem distúrbios vocais, porém falham em identificar indivíduos com distúrbio vocal. Essas medidas são aplicáveis em populações de baixo risco^(3)^, visto que apresentaram alta especificidade e AUC maior que 0.7, mas baixa sensibilidade. São elas: GNE_3000Hz_, HNRD, *jitter*_local_, *jitter*_PPQ5_,, *shimmer*_APQ3,_ *shimmer*_DDA_, SNL_2600–5100Hz_.

Destaca-se o parâmetro HNR, medida que quantifica a proporção de ruído em relação à proporção de harmônicos em uma amostra vocal, podendo ser de grande valor na diferenciação entre vozes com e sem distúrbio vocal^(28)^. No presente estudo, a medida HNR obteve o maior AUC entre as analisadas (0,837), além de uma elevada sensibilidade (90,32%), o que sugere que ela é uma das melhores métricas para detectar a presença de alterações vocais, apesar da sua especificidade moderada (64,68%) a qual indica que pode haver um número de falsos positivos. Esse fenômeno é comum em testes altamente sensíveis e deve ser considerado ao escolher a melhor abordagem diagnóstica^(27)^. Esse achado sugere que a HNR pode ser uma medida promissora para triagens iniciais onde a prioridade é identificar corretamente os casos positivos^(29)^. A combinação de medidas com alta sensibilidade (como o HNR) com outras de especificidade elevada (como o SNL 2100-5100Hz) podem otimizar a acurácia diagnóstica. Os resultados evidenciam a importância da análise acústica no diagnóstico e monitoramento da voz, ressaltando a necessidade de abordagem multimodal, combinando diferentes parâmetros para aumentar a precisão diagnóstica^(1,25)^.

A definição de pontos de corte para medidas acústicas representa um avanço importante para a prática clínica ao fornecer parâmetros objetivos que podem auxiliar na triagem e no diagnóstico diferencial entre vozes com e sem distúrbio vocal. Contudo, é fundamental destacar que a ausência de desempenho diagnóstico satisfatório para algumas medidas não implica em sua inaplicabilidade clínica. Muitas dessas medidas podem ser importantes em contextos específicos, como na comparação intra sujeito, permitindo monitorar alterações vocais ao longo do tempo, avaliar respostas a intervenções terapêuticas ou técnicas vocais, e identificar variações sutis que não são captadas por avaliações puramente categóricas. Portanto, a integração de múltiplos parâmetros acústicos, associados aos demais procedimentos da avaliação multidimensional da voz, favorece uma abordagem mais abrangente e personalizada no manejo da voz, valorizando tanto os indicadores diagnósticos robustos quanto os dados que contribuem para o acompanhamento e aprimoramento contínuo da saúde vocal^(30)^.

Todo estudo científico, por sua natureza, opera sob certas condições e escolhas metodológicas que geram limitações. O presente trabalho não é exceção, e as considerações que se seguem visam oferecer uma análise transparente desses aspectos, delineando o escopo e as fronteiras de aplicabilidade dos resultados. Contudo, embora essas limitações informem a generalização e o contexto dos achados, elas não comprometem a validade interna do estudo nem a robustez de suas principais conclusões, que permanecem relevantes e cientificamente válidas dentro do universo populacional e metodológico investigado.

O desenho transversal e descritivo impede o estabelecimento de relações causais diretas ou a avaliação da estabilidade dos pontos de corte ao longo do tempo. Adicionalmente, apesar do expressivo tamanho da amostra, o desequilíbrio entre o número de participantes com e sem distúrbio vocal, embora reflexo da realidade clínica, dificulta a generalização dos pontos de corte. Portanto, os pontos de corte desse estudo podem ser utilizados como referência na prática clínica, mas faz-se necessário estudos com amostras mais abrangentes e com populações com prevalências distintas de distúrbio vocal. A falta de um padrão-ouro reconhecido para essa finalidade pode ser considerada uma limitação do estudo. A classificação dos participantes em com e sem distúrbio vocal baseou-se na combinação do exame visual laríngeo, realizado por um médico, e do JPA realizado por fonoaudiólogos. Embora a alta confiabilidade do JPA tenha sido assegurada, seu caráter subjetivo pode influenciar a categorização. Em conjunto, essas considerações delimitam o escopo de aplicabilidade dos resultados e ressaltam a complexidade da avaliação vocal, ao mesmo tempo em que expandem e refinam o conhecimento nesta área.

Como recomendação clínica, o conjunto de medidas de eleição frente a uma suspeita de distúrbio vocal extraídas da tarefa de vogal sustentada [a] em falantes do PB são SNL_100–2600Hz,_ SNL_100–3000Hz_ SNL_100–8000Hz_, visto que apresentam sensibilidade e especificidade ≥80%. Em seguida, para fins de triagem, as medidas de eleição com sensibilidade ≥80% são GNE_1000Hz_, HNR, LNPSD, PSD, Shimmer_APQ5_, Shimmer_APQ11_ e SNL_100–5100Hz._ Finalmente, as medidas com aplicabilidade para confirmação diagnóstica com especificidade ≥80% são GNE_3000Hz_ e SNL_2600–5100Hz_.

## CONCLUSÃO

Conclui-se que 23 de um total de 44 medidas acústicas extraídas da vogal sustentada [a] de falantes do PB apresentaram métricas de desempenho satisfatório. As medidas acústicas AVI, GNE_2000Hz_, SNL_100-2600Hz_, SNL_100-3000Hz_, SNL_100-8000Hz_ e SNL_5100-8000Hz_ demonstraram capacidade discriminatória aceitável para diferenciar entre vozes com e sem distúrbio vocal. As medidas GNE_1000Hz_, HNR, LNPSD, PA, PSD,*shimmer*_APQ5,_ *shimmer*_APQ11,_ *shimmer*_dB_, *shimmer*_local,_ SNL_100–5100Hz_ apresentaram ponto de corte com sensibilidade satisfatória e podem ser utilizadas na prática clínica para fins de triagem. As medidas GNE_3000Hz,_ HNRD, Jitter _local,_Jitter _PPQ5,_ *shimmer*_APQ3,_ *shimmer*_DDA,_ SNL_2600-5100Hz_ apresentaram ponto de corte com especificidade satisfatória e possuem aplicabilidade na confirmação diagnóstica.
